# Internal resistor of multi-functional tunnel barrier for selectivity and switching uniformity in resistive random access memory

**DOI:** 10.1186/1556-276X-9-364

**Published:** 2014-07-25

**Authors:** Sangheon Lee, Jiyong Woo, Daeseok Lee, Euijun Cha, Hyunsang Hwang

**Affiliations:** 1 Department of Materials Science and Engineering, Pohang University of Science and Technology (POSTECH), Pohang 790-784, Republic of Korea

**Keywords:** ReRAM, Reliability, Selectivity

## Abstract

In this research, we analyzed the multi-functional role of a tunnel barrier that can be integrated in devices. This tunnel barrier, acting as an internal resistor, changes its resistance with applied bias. Therefore, the current flow in the devices can be controlled by a tunneling mechanism that modifies the tunnel barrier thickness for non-linearity and switching uniformity of devices. When a device is in a low-resistance state, the tunnel barrier controls the current behavior of the device because most of the bias is applied to the tunnel barrier owing to its higher resistance. Furthermore, the tunnel barrier induces uniform filament formation during set operation with the tunnel barrier controlling the current flow.

## Background

Various new types of memories, such as phase change memory, spin-torque-transfer magnetic memory, and resistive random access memory (ReRAM), have been considered to replace conventional memory owing to their improved scaling limit and low power operation [1,2]. ReRAM is the most promising candidate memory for next-generation non-volatile memory owing to the simple structure of the two-terminal type device and the fact that its cross-point array (4 F^2^) structure can be significantly scaled down. However, ReRAM exhibits large resistive-switching fluctuation and suffers from leakage current in cross-point array operation.

To mitigate the resistive switching fluctuation in ReRAM, various analyses of switching behaviors and structural solutions have been suggested [3-8]. The resistive switching uniformity is highly affected by oxide states and filament formation properties. Although various ReRAM structures have been investigated and the switching variability has been improved, ReRAMs still retain non-uniform resistive switching parameters of resistance state and voltage when the devices operate with low currents (approximately 50 μA) of devices. In addition, the currents flowing through unselected cells during the read operations are a severe problem in cross-point array ReRAMs. When a high-resistance state (HRS) cell is read, it is biased with V_Read_, while the unselected neighboring low-resistance state (LRS) cells are biased with ½V_Read_. Although LRS cells are biased with a lower voltage than the HRS cell, most current flows through the unselected LRS cells because of their very low resistance values. To prevent this sneak path current, various selection devices are introduced. Selection devices have very a high resistance at low voltage levels (V_Low_) and low resistance at high voltage levels (V_High_). Therefore, the use of a selection device and ReRAM integration can reduce the leakage current in cross-point array operation. However, they are structurally and compositionally complex for one-selector one-ReRAM (1S1R) integration [9,10]. Therefore, selector-less ReRAMs with non-linear I_LRS_ behavior and without complex compositional and structural integration have been investigated [11,12]. However, the origin of the selector-less ReRAM has not been investigated, and its switching reliability has not been considered for cross-point array operation. Most researches have focused only on the selectivity of the selector-less ReRAM.

In this research, the multi-functional role of the TiO_x_ tunnel barrier which can be integrated with ReRAM was analyzed. We significantly improved the selectivity and switching uniformity by designing the device with a simple triple-layer structure of a tunnel-barrier-layer-inserted ReRAM. The tunnel barrier can act as an internal resistor whose resistance changes with the applied bias. Direct tunneling (DT) of the tunnel barrier shows high resistance at V_Low_, whereas Fowler-Nordheim tunneling (FNT) shows low resistance at V_High_. DT of the tunnel barrier reduces the sneak-path current of the ReRAM and controls the filament formation in the HfO_2_ switching layer for selectivity and uniformity. Thus, the multi-functional tunnel barrier plays an important role in the selectivity and switching uniformity of ReRAMs.

## Experiments

We fabricated Ti/HfO_2_/multi-layer TiO_y_-TiO_x_/Pt devices in a 250-nm via-hole structure. For the isolation layer, a 100-nm-thick SiO_2_ sidewall layer was deposited on a Pt bottom electrode (BE)/Ti/SiO_2_/Si substrate by plasma-enhanced chemical vapor deposition. Subsequently, a 250-nm via-hole was formed by a KrF lithography process, followed by reactive-ion etching. First, a 6-nm TiO_x_ tunnel barrier was deposited in an Ar-and-O_2_ mixed plasma (Ar/O_2_ = 30:1 sccm) by radio frequency (RF) sputtering (working pressure 5 mTorr, RF power 100 W). To form the multi-layer TiO_y_/TiO_x_ (*y* > *x*), a tunnel barrier was annealed in O_2_ ambient by rapid thermal annealing at 300°C. We varied the thermal oxidation time to evaluate the role of the tunnel barrier in the ReRAM (0 to 10 min). Then, a switching layer of 4-nm-thick HfO_2_ was deposited using an atomic layer deposition system using TEMAH as a precursor and H_2_O as an oxidizer at 250°C. The Ti oxygen reservoir and a top electrode (TE) of 50 μm were deposited using direct current (DC) sputtering and a shadow mask.

## Discussion

**Figure 1:**
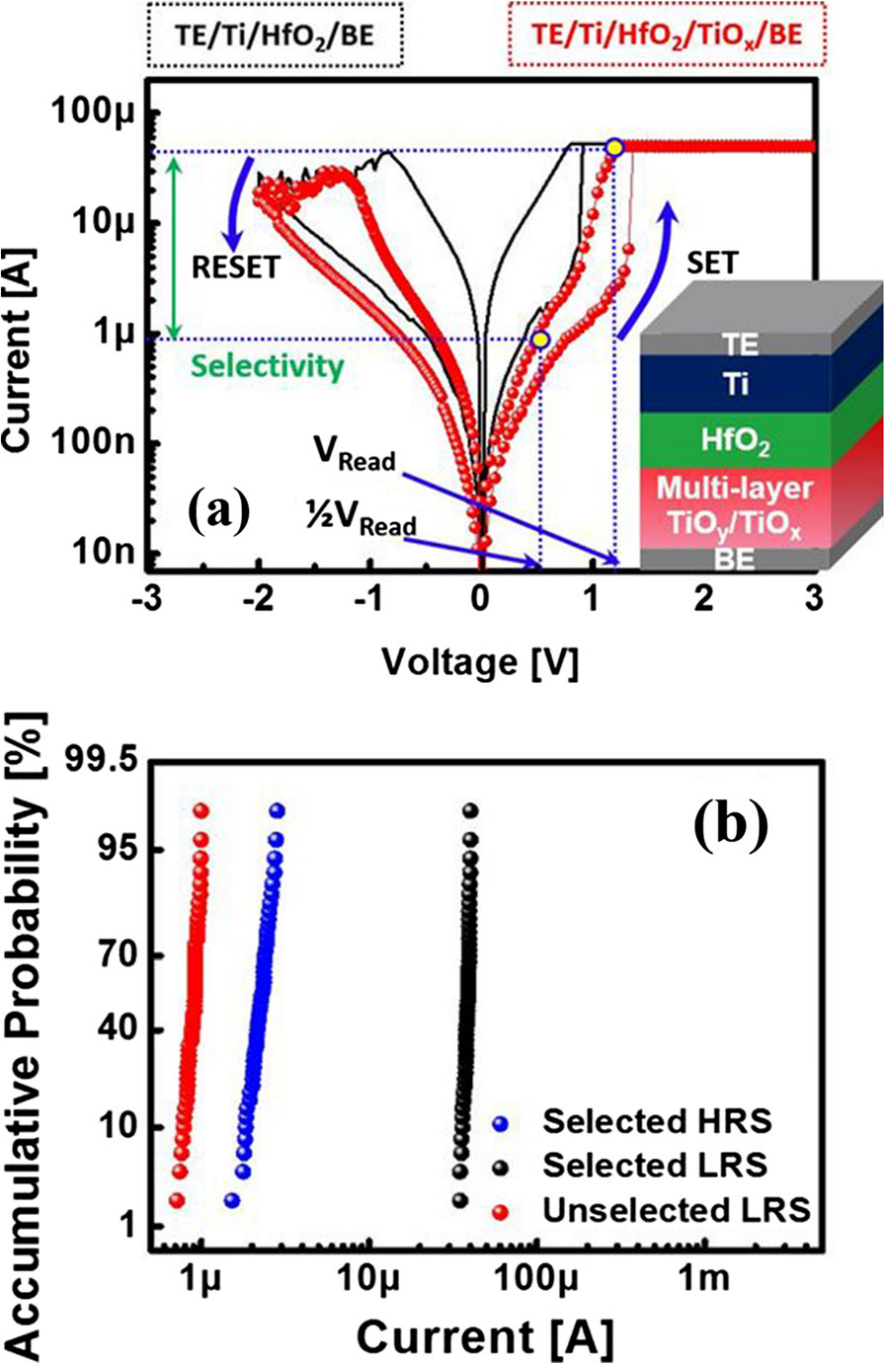
**Highly non-linear DC I-V curve and switching current distributions. (a)** Highly non-linear DC I-V curve of the selector-less ReRAM (red) and linear ReRAM (black). **(b)** Switching current (I_LRS_, black; I_HRS_, blue; and suppressed I_LRS_, red) distributions of the selector-less ReRAM.

Figure 1a shows the DC current–voltage (I-V) curve, which shows the highly non-linear I-V characteristics of the TE/Ti/HfO_2_/multi-layer TiO_y_-TiO_x_/BE device. A 50-μA compliance current was used to prevent hard breakdown. A DC bias was applied to the TE, and the BE was grounded. To induce oxygen vacancy (V_o_) filament formation during the set operation, a positive bias was applied to the TE. In contrast, a negative bias was applied to the TE to dissolve the filament. For the reading operation, V_Read_ (1.1 V) was applied to the selected cells while ½V_Read_ (0.55 V) was applied to the unselected cells in the cross-point array. Thus, the sneak-path current of V_Low_ should be significantly suppressed. We observed that I_LRS_ was greatly suppressed at ½V_Read_ with high selectivity (Figure 1a). To confirm the switching reliability of the selector-less ReRAM, switching current distributions were calculated. As shown in Figure 1b, this device exhibited highly reliable resistance switching. Furthermore, the I_LRS_ at ½V_Read_ was sufficiently suppressed, making it usable for cross-point array applications.

In the device structure shown in Figure 1a, Ti/HfO_2_ acts as a memory with filament formation and dissolution with set and reset operations. The integrated multi-layer TiO_y_/TiO_x_ acts as an internal resistor for the non-linear I_LRS_ and the filament formation control. Accordingly, the memory and multi-layer tunnel barrier can be considered as serially connected resistors. Thus, if the operating current of the ReRAM is higher than that of the internal resistor (R_ReRAM_ < R_internal resistor_), the current of the ReRAM is mainly determined by the internal resistor. In serially connected resistors, most of the bias is applied to the higher resistance, and the same current flows through the lower resistance. Therefore, we analyzed the behaviors of the selector-less ReRAM, which is integrated with the internal resistor of the TiO_x_ tunnel barrier.

**Figure 2:**
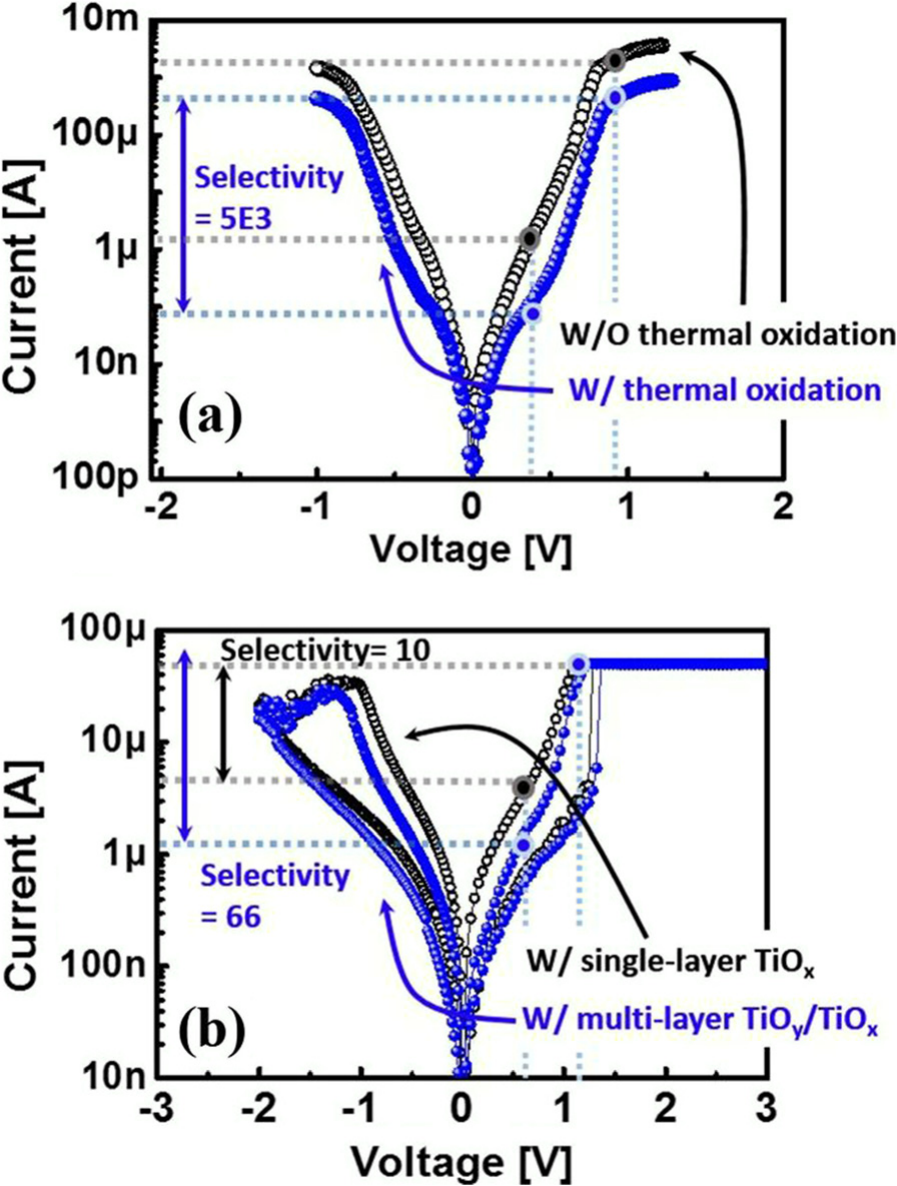
**DC I-V and non-linear behavior comparisons. (a)** DC I-V comparison of multi-layer tunnel barrier (blue) and single-layer tunnel barrier (black). **(b)** Comparison of the non-linear behaviors of the selector-less ReRAMs by inserting multi-layer (blue) and single-layer tunnel barriers (black).

First, it is well known that the tunnel barrier can exhibit non-linear I-V characteristics owing to the electric-field-controlled modification of the barrier thickness of the tunnel barrier [12,13]. The modification of the barrier thickness of the tunnel barrier exhibits DT and FNT for suppressed current and sufficient current at V_Low_ and V_High_, respectively. To increase the effect of DT on I_LRS_ at ½V_Read_, we carried out thermal oxidation of the TiO_x_ tunnel barrier layer to form more insulating TiO_y_ (*y* > *x*) on the top surface of TiO_x_ in the multi-layer TiO_y_/TiO_x_. To study the role of the tunnel barrier in selectivity, we fabricated and evaluated Pt/multi-layer TiO_y_-TiO_x_/Pt and Pt/single-layer TiO_x_/Pt structures. Neither the multi-layer nor the single-layer tunnel barriers exhibited hysteric behaviors, as shown in Figure 2a. The multi-layer TiO_y_/TiO_x_ exhibited higher selectivity of the internal resistor than the single-layer TiO_x_ (Figure 2a). We confirmed that the tunnel barrier can act as an internal resistor that has variable resistance for non-linear I_LRS_ of the device. The selectivity of the tunnel barrier internal resistor was dependent on the thermal oxidation time of the TiO_x_ tunnel barrier. Higher selectivity was observed in the multi-layer TiO_y_/TiO_x_ than in the single-layer TiO_x_ without thermal oxidation. TiO_y_ can suppress electron transfer more than TiO_x_ at V_Low_ because of its more insulating state. Once a filament is formed in the HfO_2_ switching layer, the tunnel barrier dominantly is the dominant factor that controls I-V characteristics with barrier thickness modification because R_LRS_ is much lower than R_tunnel barrier_. Therefore, it was observed that the high non-linear I_LRS_ of the ReRAM could be achieved by inserting a multi-layer tunnel barrier (Figure 2b). The non-linearity of the selector-less ReRAM was higher in the multi-layer tunnel barrier than that of the single-layer tunnel barrier.

**Figure 3:**
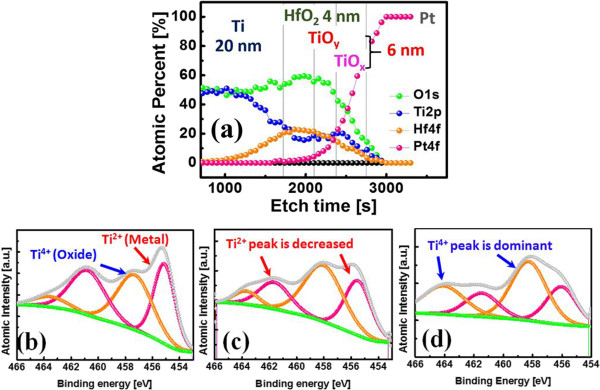
**Depth profile and bonding energy change. (a)** Depth profile of the selector-less ReRAM. **(b, c, d)** Bonding energy change in the TiO_x_ top surface with thermal oxidation time (0-, 5-, and 10-min oxidation). Ti^4+^ peak increased with increasing thermal oxidation time.

Figure 3 shows the depth profile of the device and the tendency of the TiO_x_ top surface bonding energy in relation to the thermal oxidation time. Figure 3a shows the depth profile of the selector-less ReRAM to confirm the device structure. Every depth point was detected with an etching rate of 3 min. Total etch time to detect BE of Pt was 34 min. Figure 3b, c, d shows the bonding energy of the multi-layer TiO_y_/TiO_x_ tunnel barrier. We focused on the top surface of the TiO_x_ layer to confirm the thermal oxidation effect. By increasing the thermal oxidation time, we observed that the Ti^4+^ peak of the insulating TiO_x_ phase increases because of thermal oxidation. In addition, the Ti^2+^ peak of metal Ti relatively decreases owing to thermal oxidation. Therefore, it can be seen that the multi-layer TiO_y_/TiO_x_ exhibits highly non-linear behavior owing to excellent tunnel barrier characteristics (Figure 2a,b).

**Figure 4:**
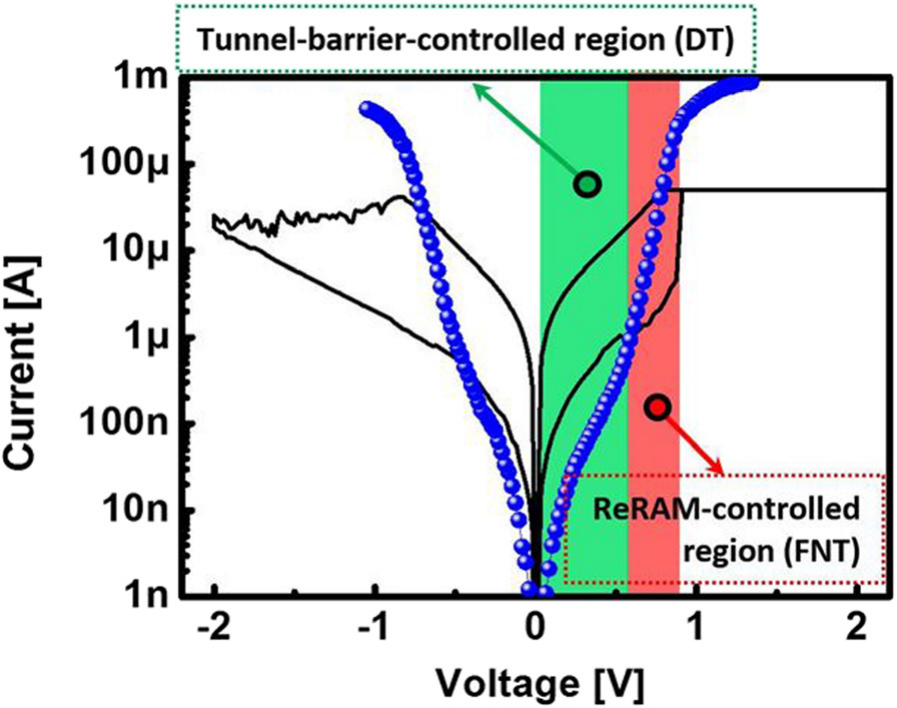
Comparison of ReRAM (black) and tunnel barrier (blue) DC I-V curves.

**Figure 5:**
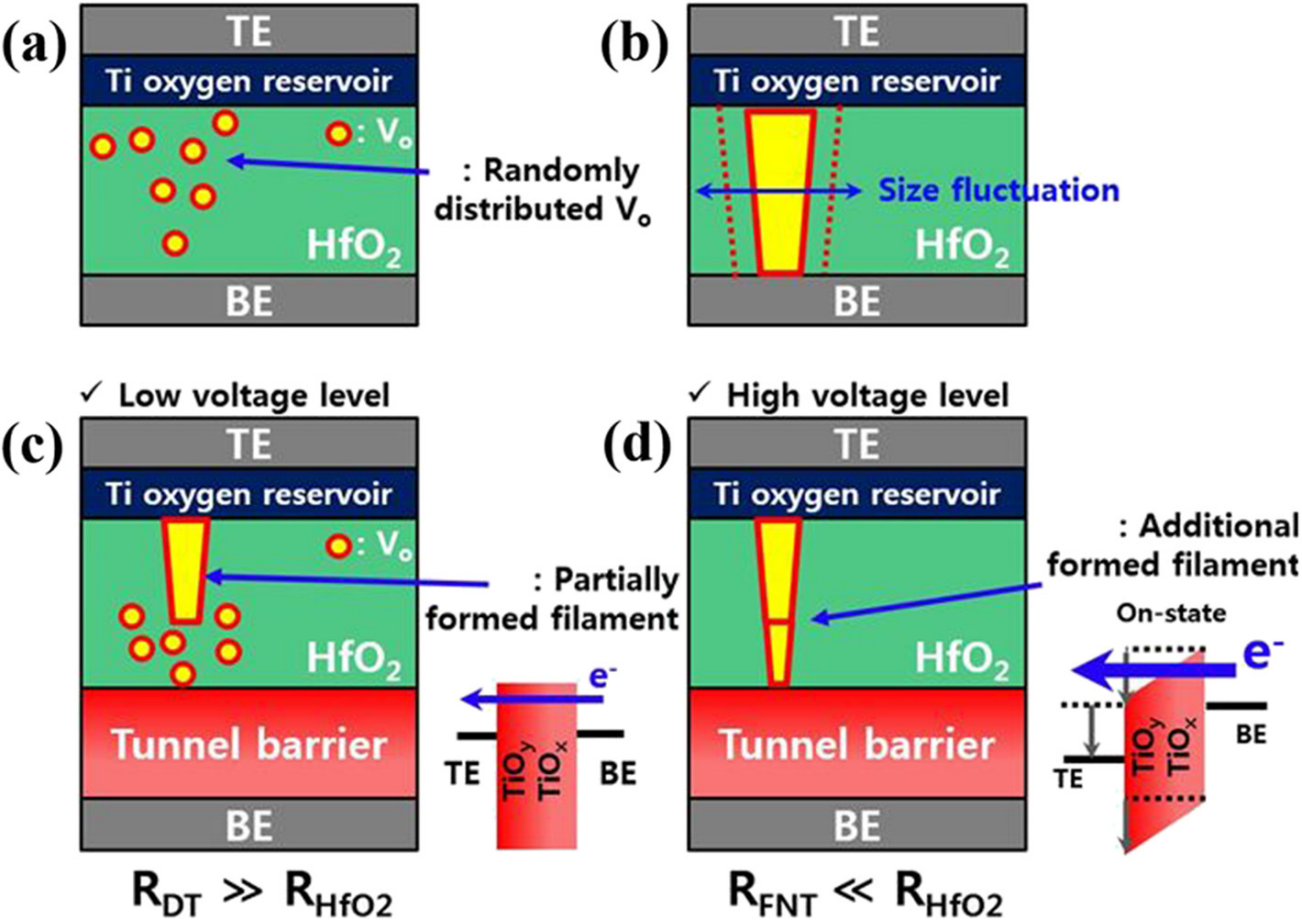
Concept of filament formation in general ReRAM (a, b) and the selector-less ReRAM (c, d).

Second, the tunnel barrier controls filament formation during the set operation for uniform resistive switching. In general, the filament size of the ReRAM can have random fluctuation owing to the randomly distributed oxygen vacancy (V_o_) of binary metal oxide switching layers and the uncontrollable current flowing during the set operation. Furthermore, a fluctuating filament reflects the large fluctuation of the reset operation, and it results in large fluctuation of HRS distributions. In the ReRAM operating mechanism, randomly distributed V_o_ is an intrinsic characteristic of the ReRAM. To achieve uniform switching behavior, the current flowing in the switching layer should be controlled. Therefore, the insertion of additional layers, such as filament formation control layers, has been investigated for the control of current flow. It is well known that internal resistors or external resistors can induce reliable filament formation with the controlled current flowing through serially connected resistors [14,15]. When compared to linear resistors, the tunnel barrier can be considered as a non-linear resistor. The resistance of this multi-layer tunnel barrier can vary with the applied bias owing to tunnel barrier thickness modification. The resistance of the tunnel barrier is very high at the DT-controlled bias level, whereas the resistance of the tunnel barrier is very low at the FNT-controlled bias level. The resistance of a typical ReRAM can be determined by the filament growth rate. Thus, the tunnel-barrier-integrated ReRAM can be considered to comprise a serially connected switching layer resistance (R_HfO2_) and tunnel barrier resistance (R_Tunnel barrier_). R_HfO2_ can be changed to R_HRS_, an intermediate resistance state (R_IRS_), and R_LRS_ with filament growth thickness. The R_HfO2_ value decreases with filament growth. In the case of the multi-layer tunnel barrier, the resistances can be considered as a DT resistance (R_DT_) and FNT resistance (R_FNT_) at V_Low_ and V_High_, respectively. Accordingly, the dominant layer changes with the resistance values. Figure 4 compares the DC I-V curves of the multi-layer tunnel barrier and linear ReRAM. At V_Low_, the operating current of the tunnel barrier is much lower than that of the ReRAM HRS. In contrast, the operating current of the tunnel barrier is much higher than that of the ReRAM HRS at V_High_. Therefore, the tunnel barrier is dominant at V_Low_, and the ReRAM is dominant at V_High_ in the ReRAM HRS. Figure 5 shows the concept of filament formation during the set operation of a linear ReRAM and the selector-less ReRAM. As shown in Figure 5c,d, most bias is applied to the tunnel barrier owing to R_DT_ > R_HRS_ at V_Low_. During the positive bias increase for filament formation, V_o_s are cohesive, and a partial filament is formed with the tunnel barrier controlled current until the dominant region changes (Figures 4 and 5c). Accordingly, the filament size may be relatively smaller than that of linear ReRAMs owing to the suppressed current flow. When less current flows along the device, smaller filament is formed. Therefore, partial filament formation is achieved with R_DT_ (Figure 5c).

**Figure 6:**
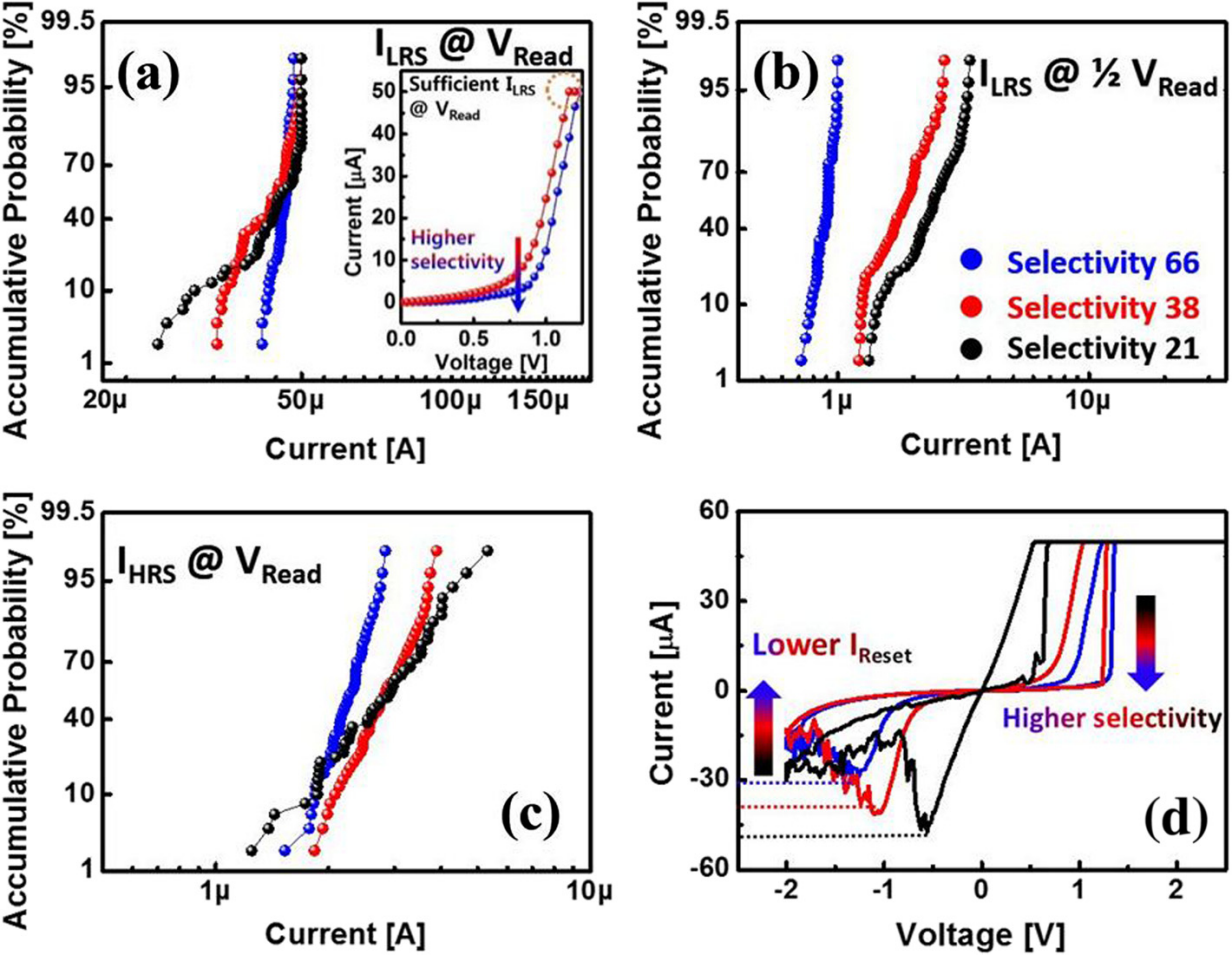
**Switching current distributions (a, b, c) and relationship between selectivity values and I**_**Reset **_**(d). (a, b, c)** Switching current distributions with various tunnel barriers with various selectivity values (selectivity of blue, red, and black are 66, 38, and 21, respectively). **(d)** Relationship between selectivity values and I_Reset_.

The partial filament state can be considered as an IRS, which is R_LRS_ < R_IRS_ < R_HRS_. At V_High_, the tunnel barrier is lowered and most bias is applied to the ReRAM owing to R_IRS_ > R_FNT_ (Figure 2). Thus, filament formation is determined by the intrinsic ReRAM characteristics without any influence of the tunnel barrier. An additional filament can be formed along the partially formed filament for achieving set operation of the LRS because most of the electric field and current focus on the partially formed conductive filament path (Figure 5d). Consequently, the tunnel-barrier-integrated ReRAM can exhibit higher switching uniformity than a control sample without a tunnel barrier. Furthermore, the selected LRS and HRS and unselected LRS switching current uniformity were more reliable with the higher selectivity of the ReRAM, which has the multi-layer TiO_y_/TiO_x_, than with the lower selectivity of the ReRAM (Figure 6a,b,c). We confirmed that resistive switching uniformity can be improved by a tunnel barrier of high selectivity. In the case of higher selectivity, the R_DT_ value is higher and more effectively controls the current flow of the ReRAM for uniform small filament formation. The smaller filament formation with higher selectivity was confirmed by the lower reset current (I_Reset_), as shown in Figure 6d. In general, I_Reset_ is related to filament size, and a larger filament requires a higher I_Reset_. It is well known that the filament size is determined at the set operation, and the filament size determines I_Reset_[16,17]. Thus, a higher selectivity of the ReRAM leads to a lower I_Reset_ with smaller filament formation by tunnel barrier controlled current flow.

**Figure 7:**
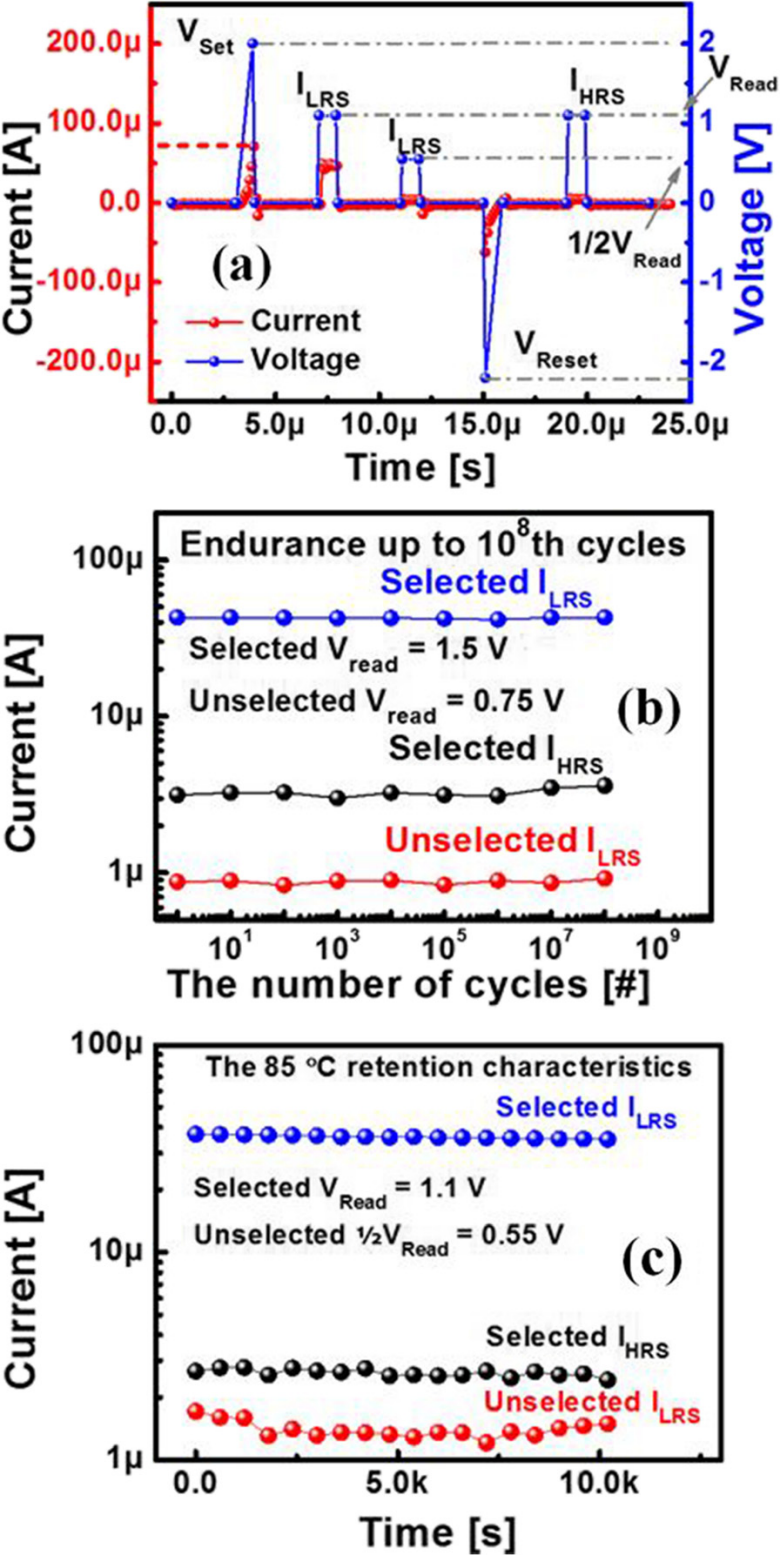
Pulse conditions (a), endurance reliability (b), and retention (c) measurement.

Finally, the reliability of non-volatile memory applications was evaluated. To measure endurance, we applied a 1-μs pulse width of +2 V/-2.2 V (Figure 7a). It exhibited high endurance of up to 10^8^ cycles (Figure 7b). Furthermore, we confirmed that the selector-less ReRAM suppressed leakage current in AC pulse operation. In a real cross-point array, pulse operation characteristics are highly important. In addition, retention was measured at 85°C for more than 10^4^ s without noticeable degradation (Figure 7c).

## Conclusion

The role of a multi-functional tunnel barrier was investigated. The main concern areas of selectivity and switching uniformity were significantly improved. This is attributed to the tunnel barrier acting as an internal resistor that controls electron transfer owing to its variable resistance. In addition, the effect of the tunnel barrier on selectivity and switching uniformity was stronger in a multi-layer TiO_y_/TiO_x_ than in a single-layer TiO_x_ owing to the greater suppression of the V_Low_ current flow.

## Competing interests

The authors declare that they have no competing interests.

## Authors’ contributions

SL had studied and analyzed behaviors of resistive random access memory (ReRAM) for high selectivity and switching uniformity. He observed that the TiO_x_ tunnel barrier plays an important role in selectivity and switching uniformity. Firstly, JW observed the non-linear behavior of the ReRAM in our group. DL participated in the switching uniformity analysis. EC participated in the study of the filament growth. Prof. HH comprehensively understands this work as an advisor. All authors have read and approved the final manuscript.

## Authors’ information

SL is currently a 2nd-year Ph.D. candidate at the Materials Science and Engineering of POSTECH, and his research field is ReRAM process and integration for high density memory.
